# High pH-Sensitive Store-Operated Ca^2+^ Entry Mediated by Ca^2+^ Release-Activated Ca^2+^ Channels in Rat Odontoblasts

**DOI:** 10.3389/fphys.2018.00443

**Published:** 2018-05-01

**Authors:** Maki Kimura, Koichi Nishi, Asuka Higashikawa, Sadao Ohyama, Kaoru Sakurai, Masakazu Tazaki, Yoshiyuki Shibukawa

**Affiliations:** ^1^Department of Physiology, Tokyo Dental College, Tokyo, Japan; ^2^Department of Removable Prosthodontics and Gerodontology, Tokyo Dental College, Tokyo, Japan; ^3^Department of Oral Surgery, Tokyo Metropolitan Cancer and Infectious Diseases Center Komagome Hospital, Tokyo, Japan

**Keywords:** odontoblast, store-operated Ca^2+^ entry, Ca^2+^ release-activated Ca^2+^ channel, alkaline stimulation, dentinogenesis

## Abstract

Odontoblasts play a crucial role in dentin formation and sensory transduction following the application of stimuli to the dentin surface. Various exogenous and endogenous stimuli elicit an increase in the intracellular free calcium concentration ([Ca^2+^]_i_) in odontoblasts, which is mediated by Ca^2+^ release from intracellular Ca^2+^ stores and/or Ca^2+^ influx from the extracellular medium. In a previous study, we demonstrated that the depletion of Ca^2+^ stores in odontoblasts activated store-operated Ca^2+^ entry (SOCE), a Ca^2+^ influx pathway. However, the precise biophysical and pharmacological properties of SOCE in odontoblasts have remained unclear. In the present study, we examined the functional expression and pharmacological properties of Ca^2+^ release-activated Ca^2+^ (CRAC) channels that mediate SOCE and evaluated the alkali sensitivity of SOCE in rat odontoblasts. In the absence of extracellular Ca^2+^, treatment with thapsigargin (TG), a sarco/endoplasmic reticulum Ca^2+^-ATPase inhibitor, induced an increase in [Ca^2+^]_i_. After [Ca^2+^]_i_ returned to near-resting levels, the subsequent application of 2.5 mM extracellular Ca^2+^ resulted in an increase in [Ca^2+^]_i_ which is a typical of SOCE activation. Additionally, application of 2-methylthioadenosine diphosphate trisodium salt (2-MeSADP), a P2Y_1_,_12_,_13_ receptor agonist, or carbachol (CCh), a muscarinic cholinergic receptor agonist, in the absence of extracellular Ca^2+^, induced a transient increase in [Ca^2+^]_i_. The subsequent addition of extracellular Ca^2+^ resulted in significantly higher [Ca^2+^]_i_ in 2-MeSADP- or CCh-treated odontoblasts than in untreated cells. SOCE, that is activated by addition of extracellular Ca^2+^ in the TG pretreated odontoblasts was then suppressed by Synta66, BTP2, or lanthanum, which are CRAC channel inhibitors. Treatment with an alkaline solution enhanced SOCE, while treatment with HC030031, a TRPA1 channel antagonist, inhibited it. The amplitude of SOCE at pH 9 in the presence of HC030031 was higher than that at pH 7.4 in the absence of HC030031. These findings indicate that CRAC channel-mediated alkali-sensitive SOCE occurs in odontoblasts. SOCE is mediated by P2Y and muscarinic-cholinergic receptors, which are activated by endogenous ligands in odontoblasts.

## Introduction

Along with their role in physiological dentin formation and mineralization (dentinogenesis), odontoblasts are important players in sensory transduction following various stimuli to the dentin surface (Linde, 1995; Linde and Lundgren, 1995; Tsumura et al., 2012, 2013; Sato et al., 2013, 2015; Shibukawa et al., 2015; Kimura et al., 2016; Nishiyama et al., 2016). The stimuli to the dentin surface induce Ca^2+^ signaling, resulting in increased intracellular free Ca^2+^ concentration ([Ca^2+^]_i_) in odontoblasts, which triggers the release of ATP from pannexin channels, and glutamate from the volume-sensitive outwardly rectifying anion channels as neuro-/intercellular-transmitters (Sato et al., 2015; Shibukawa et al., 2015; Nishiyama et al., 2016). The released ATP and glutamate play a role in the sensory signal transduction sequence for dentinal pain by transmitting sensory signals to neurons, and may promote dentinogenesis by a cluster of odontoblasts via odontoblast-odontoblast and odontoblast-trigeminal ganglion (TG) neuron signal communication (Sato et al., 2015; Shibukawa et al., 2015; Nishiyama et al., 2016). In addition, we also reported that high pH-sensitive transient receptor potential (TRP) ankyrin subfamily member 1 (TRPA1) channel activation facilitates dentinogenesis in odontoblasts in an external high pH environment (Kimura et al., 2016).

In odontoblasts, Ca^2+^ signaling is mediated by two closely related components: external stimuli-evoked Ca^2+^ influx from the extracellular medium and Ca^2+^ release from intracellular Ca^2+^ stores. Ca^2+^ influx is caused by the activation of TRP channels following the generation of external stimuli-induced hydrodynamic forces inside the dentinal tubes (Tsumura et al., 2012, 2013; Sato et al., 2013, 2015; Shibukawa et al., 2015). Ca^2+^ release from intracellular Ca^2+^ stores is mediated by inositol-1, 4, 5-triphosphate (IP_3_) receptors or ryanodine receptors in response to G-protein coupled receptor (GPCR) activation or depolarization (Shibukawa and Suzuki, 1997, 2003). It has been shown that GPCRs expressed in odontoblasts are activated by endogenous ligands, such as ATP and glutamate released from odontoblasts as well as acetylcholine, and bradykinin (Shibukawa and Suzuki, 2003; Ichikawa et al., 2012; Sato et al., 2015; Shibukawa et al., 2015; Nishiyama et al., 2016). Activation of GPCRs by binding of these ligands relays the signal to the Gα_q_ family, and stimulates phospholipase C to degrade phosphatidylinositol 4, 5-bisphosphate into IP_3_, and membrane-bound diacylglycerol. IP_3_ then activates the Ca^2+^ permeable IP_3_ receptor channels on the Ca^2+^ stores (Rhee and Bae, 1997; Syrovatkina et al., 2016). Thus, both Ca^2+^ influx from extracellular medium and Ca^2+^ release from Ca^2+^ stores increase [Ca^2+^]_i_, and the increased intracellular Ca^2+^ is extruded to the extracellular medium via Na^+^-Ca^2+^ exchanger (NCX) subtypes 1, and 3 (Lundgren and Linde, 1988; Lundquist et al., 2000; Tsumura et al., 2010), and/or Ca^2+^–ATPase (PMCA) (Linde and Lundgren, 1995) in the distal end of plasma membrane in odontoblasts. This Ca^2+^ extrusion to the dentin-mineralizing front is involved in dentinogenesis. The increased intracellular Ca^2+^ is also taken up into the Ca^2+^ stores via sarco-endoplasmic reticulum Ca^2+^–ATPase (SERCA) (refilling) (Lundgren and Linde, 1997).

Store-operated Ca^2+^ entry (SOCE) has been well described as a Ca^2+^ entry pathway in the plasma membrane that is activated by the depletion of IP_3_- and/or ryanodine-sensitive Ca^2+^ stores (Putney, 1986, 2010; Parekh and Putney, 2005). SOCE is a ubiquitous and important Ca^2+^ influx mechanism in excitable and non-excitable cells. SOCE participates not only in the replenishment of Ca^2+^ stores but also in the modulation of many physiological functions such as secretion, cell proliferation, endothelial cell migration, T cell activation, mast cell degranulation, thrombus formation, and tumor cell metastasis (Cheng et al., 2011). SOCE is mediated via store-operated Ca^2+^ (SOC) channels. The best-characterized SOC channels are Ca^2+^ release-activated Ca^2+^ (CRAC) channels composed of the pore-forming subunit Orai1, Orai2, or Orai3 (Desai et al., 2015). The depletion of Ca^2+^ stores is sensed by Ca^2+^ store-localized stromal interaction molecule 1 (STIM1), a Ca^2+^ store calcium-sensor, and causes translocation of STIM1 to the plasma membrane. In the plasma membrane, STIM1 interacts directly with Orai1, resulting in the activation of CRAC channels (Frischauf et al., 2008, 2016; Desai et al., 2015; Desvignes et al., 2015). Recent studies have shown the importance of Orai1 in bone formation by osteoblasts. Both odontoblasts and osteoblasts evoke the secretion of the extracellular matrix and formation of mineralized hydroxyapatite (Hwang et al., 2012). In addition, in ameloblasts, SOCE contributes to enamel formation and regulation of the expression of enamel matrix proteins. CRAC channels are also involved in enamel development (Nurbaeva et al., 2015b).

It has been reported that Orai1 is expressed in mouse odontoblasts (Zheng et al., 2015). In a previous study, we had also demonstrated that SOCE and CRAC currents were activated in response to depletion of Ca^2+^ stores in acutely dissociated odontoblasts (Shibukawa and Suzuki, 2003). However, the detailed biophysical as well as pharmacological properties of SOCE in odontoblasts remain unclear. In the present study, to elucidate pharmacological properties of SOCE, we investigated the expression, and pharmacological properties of CRAC channels in odontoblasts. In addition, we examined extracellular pH-sensitivity of the SOCE in odontoblasts.

## Materials and Methods

### Ethical Approval

All animals were treated in accordance with the Guiding Principles for the Care and Use of Animals in the field of physiological sciences approved by the Council of the Physiological Society of Japan and the American Physiological Society. All animal experiments in this study followed the guidelines established by the National Institutes of Health, United States regarding the care and use of animals for experimental procedures, as well as the United Kingdom Animals (Scientific Procedures) Act, 1986. This study was approved by the Ethics Committee of our institute (Nos. 270302, 280302, and 290301).

### Dental Pulp Slice Preparation

Dental pulp slice preparations were obtained from newborn Wistar rats (6- to 8-day-old) using a previously described method (Son et al., 2009; Shibukawa et al., 2015; Tokuda et al., 2015). Briefly, under isoflurane (3%) and pentobarbital sodium anesthesia (25 mg/kg), the mandible was dissected. The hemimandible embedded in alginate impression material was sliced transversely through the incisor at 500-μm thickness with a standard vibrating tissue slicer (Dosaka EM, Kyoto, Japan). A section of mandible was sliced to the required level, so that the dentin and enamel were directly visible between the bone tissue and the dental pulp. The surrounding impression material, bone tissue, enamel, and dentin were removed from the mandible section under a stereoscopic microscope, and the remaining dental pulp slice was used in further experiments. We selected mandible sections in which the dentin layer was thin and the enamel, and dentin were clearly distinguishable under the microscope, to avoid cellular damage to odontoblasts. Pulp slices were treated with a standard Krebs solution containing 0.03% trypsin and 0.17% collagenase (30 min at 37°C). For [Ca^2+^]_i_ measurement, enzymatically treated and isolated odontoblasts from the dental pulp slice were plated onto a culture dish, immersed in alpha-minimum essential medium (Life Technologies, Carlsbad, CA, United States) including 10% fetal bovine serum and 5% horse serum, and maintained at 37°C in a 5% CO_2_ incubator. The primary cultured odontoblasts from the dental pulp slice were used for [Ca^2+^]_i_ measurements; we stably measured [Ca^2+^]_i_ increases within 24 h of isolation. Cells were confirmed to be odontoblasts in a previous study with the odontoblast markers dentin matrix protein-1, dentin sialoprotein, and nestin within 24 h of isolation (Tsumura et al., 2012).

### Measurement of Ca^2+^-Sensitive Dye Fluorescence

Cells in dental pulp slices were loaded with 10 μM fura-2-acetoxymethyl ester (Dojindo Laboratories, Kumamoto, Japan) (Tsien et al., 1985) and 0.1% (w/v) pluronic acid F-127 (Life Technologies) in standard Krebs solution for 30 min at 37°C. They were then washed with fresh Krebs solution. A dish including fura-2-loaded odontoblasts was mounted on the stage of a microscope (IX73, Olympus, Tokyo, Japan) with HCImage software, an excitation wavelength selector, and an intensified charge-coupled device camera system (Hamamatsu Photonics, Shizuoka, Japan). Fura-2 fluorescence emission was recorded at 510 nm in response to alternating excitation wavelengths of 380 nm (F380) and 340 nm (F340). The [Ca^2+^]_i_ was defined using the fluorescence ratio (*R*_F340/F380_) of F340 to F380 (F340/F380), and is described as *F*/*F*_0_ units; the *R*_F340/F380_ value (*F*) was normalized to the resting value (*F*_0_). The *F*/*F*_0_ baseline was configured at 1.0. All experiments were performed at room temperature (30 ± 1.0°C).

### Solutions and Reagents

Krebs solution containing (in mM) 136 NaCl, 5 KCl, 0 or 2.5 CaCl_2_, 0.5 MgCl_2_, 10 HEPES, 10 glucose, and 12 NaHCO_3_ (pH 7.4 Tris) was used as the standard extracellular solution. To prepare high pH (pH 9) extracellular solutions, 12 mM NaHCO_3_ in Krebs solution was replaced by 10 mM (for pH 9) NaOH. This replacement did not affect extracellular free Ca^2+^ concentrations in the test solution. Synta66 was obtained from AOBIOUS INC. (Gloucester, MA, United States). BTP2 was obtained from Santa Cruz Biotechnology (Santa Cruz, CA, United States). HC030031, 2-Methylthioadenosine diphosphate, and DHPG were obtained from Tocris Bioscience (Bristol, United Kingdom). All other reagents were obtained from Sigma Chemical Co. (St. Louis, MO, United States). Stock solutions of lanthanum chloride, carbachol, and DHPG were prepared in ultra-pure water (Millipore, MA, United States). All other stock solutions were prepared in dimethyl sulfoxide. Stock solutions were diluted to the appropriate concentration with Krebs solution (pH 7.4 or 9) before use.

### Statistics and Offline Analysis

Data are represented as the mean ± standard error (SE) of the mean of *N* observations, where *N* shows the number of independent experiments. The Wilcoxon test or Mann–Whitney test were used to evaluate the non-parametric statistical significance. A *P*-value < 0.05 was considered significant. Statistical analysis was performed using GraphPad Prism 7.0 (GraphPad Software, La Jolla, CA, United States).

## Results

### Addition of Extracellular Ca^2+^ Following Ca^2+^ Store Depletion Increased [Ca^2+^]_i_

**FIGURE 1:**
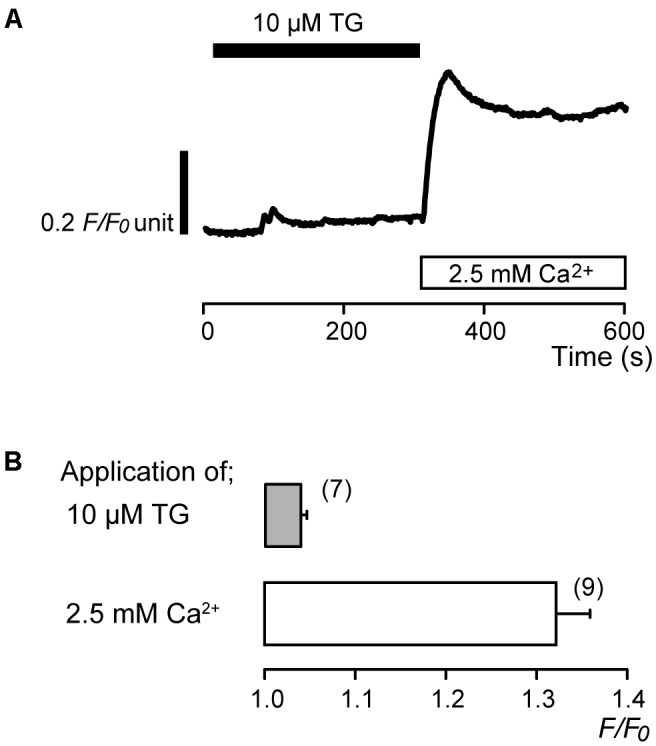
Addition of extracellular Ca^2+^ increases [Ca^2+^]_i_ following TG-induced [Ca^2+^]_i_ increase. **(A)** Representative trace of [Ca^2+^]_i_ increase in response to application of 10 μM TG and subsequent application of 2.5 mM extracellular Ca^2+^ (white box at bottom) after 10 μM TG application. Black box at the top indicates the application of 10 μM TG. **(B)** Summary bar graph shows [Ca^2+^]_i_ increases by application of 10 μM TG (gray column) and 2.5 mM extracellular Ca^2+^ (open column). Each column indicates the mean ± SE of 7–9 independent experiments.

In the absence of extracellular Ca^2+^, application of 10 μM thapsigargin (TG), an inhibitor of sarco/endoplasmic reticulum Ca^2+^-ATPase (Thastrup et al., 1990; Shibukawa and Suzuki, 2003), induced transient [Ca^2+^]_i_ increases to a peak value of 1.04 ± 0.006 *F*/*F*_0_ units (*N* = 7) (**Figures 1A,B**). The transient increases in [Ca^2+^]_i_ are caused by the release of Ca^2+^ from intracellular Ca^2+^ stores. After [Ca^2+^]_i_ returned to the near-resting levels, subsequent application of 2.5 mM extracellular Ca^2+^ increased [Ca^2+^]_i_ (**Figure 1A**) to a peak value of 1.32 ± 0.04 *F*/*F*_0_ units (*N* = 9) (**Figures 1A,B**).

### Effects of 2-MeSADP, Carbachol and DHPG Pre-application on the Ca^2+^ Influx

**FIGURE 2:**
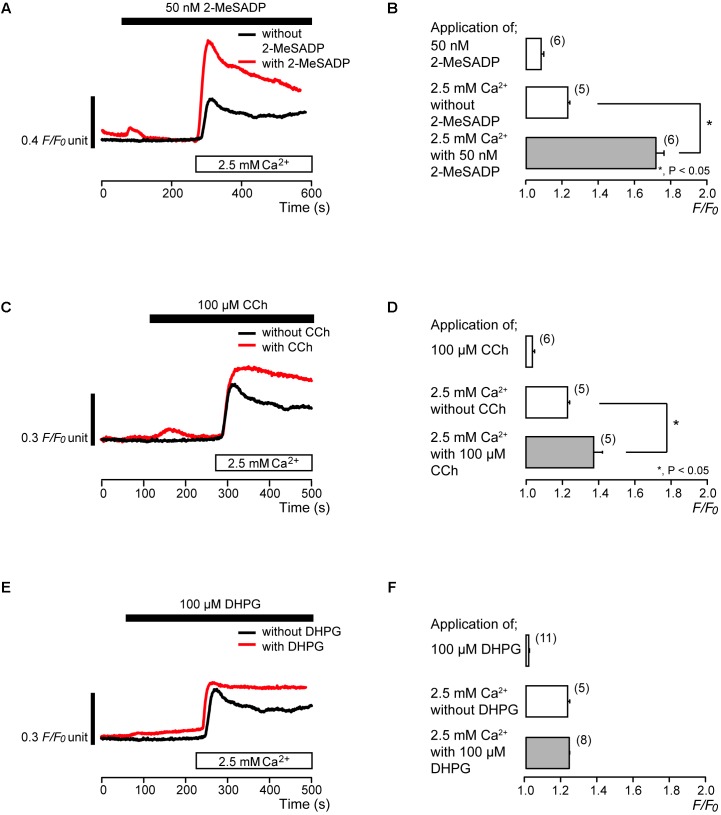
Effects of PLC-coupled receptor agonists on the Ca^2+^ influx. **(A,C,E)** Representative traces of [Ca^2+^]_i_ increase in response to application of PLC-coupled receptor agonists, 50 nM 2-MeSADP **(A)**, 100 μM CCh **(C)**, or 100 μM DHPG **(E)**, and subsequent application of 2.5 mM extracellular Ca^2+^ (white box at bottom) with (red line), or without (black line) agonists **(A,C,E)**. Black boxes at the top indicate the application time period of 50 nM 2-MeSADP **(A)**, 100 μM CCh **(C)**, or 100 μM DHPG **(E)**. **(B,D,F)** Summary bar graphs show [Ca^2+^]_i_ increase by application of 50 nM 2-MeSADP **(B)**, 100 μM CCh **(D)**, or 100 μM DHPG **(F)** (upper column) and subsequent application of 2.5 mM extracellular Ca^2+^ in the presence (gray column) or absence (middle column) of these agonists. Each column indicates the mean ± SE of 5–11 independent experiments. Statistically significant differences between columns (shown by solid lines) are denoted by asterisks, ^∗^*P* < 0.05.

PLC-coupled receptors, P2Y (Sato et al., 2015; Shibukawa et al., 2015; Wang et al., 2016), muscarinic-cholinergic (Shibukawa and Suzuki, 2003), and group I metabotropic glutamate receptors (Kim et al., 2009; Nishiyama et al., 2016), are expressed in odontoblasts. We, thus, examined the participation of these PLC-coupled receptors in the activation of Ca^2+^ influx by store depletion. In the absence of extracellular Ca^2+^, application of 50 nM 2-methylthioadenosine diphosphate (2-MeSADP), a P2Y_1,12,13_ receptor agonist (Abbracchio et al., 2006; Kawaguchi et al., 2015), increased [Ca^2+^]_i_ transiently to a peak value of 1.08 ± 0.02 *F*/*F*_0_ units (*N* = 6) (**Figures 2A,B**). Carbachol (CCh) (100 μM), a muscarinic-cholinergic receptor agonist (He et al., 2005; Piergentili et al., 2007), evoked transient [Ca^2+^]_i_ increases to the value of 1.04 ± 0.01 *F*/*F*_0_ units (*N* = 6) (**Figures 2C,D**), while application of 100 μM DHPG, an agonist of group I metabotropic glutamate receptors (Ito et al., 1992; Lin et al., 1997; Schoepp et al., 1999), induced transient [Ca^2+^]_i_ increases to the value of 1.02 ± 0.002 *F*/*F*_0_ units (*N* = 11) (**Figures 2E,F**). These transient [Ca^2+^]_i_ increases are elicited by the Ca^2+^ release from intracellular Ca^2+^ stores. After [Ca^2+^]_i_ returned to near-resting levels following each application of 50 nM 2-MeSADP, 100 μM CCh, and 100 μM DHPG, subsequent addition of 2.5 mM extracellular Ca^2+^ increased [Ca^2+^]_i_ (**Figures 2A,C,E**). The peak values following application of 2.5 mM extracellular Ca^2+^ with 50 nM 2-MeSADP were 1.72 ± 0.04 *F*/*F*_0_ units (*N* = 6) (**Figure 2B**), while those with 100 μM CCh were 1.38 ± 0.05 *F*/*F*_0_ units (*N* = 5) (**Figure 2D**). After pretreatment of 2-MeSADP, and CCh, the Ca^2+^ influx induced by subsequent application of 2.5 mM extracellular Ca^2+^ was significantly larger than that without pretreatment; the values of Ca^2+^ influx without any pretreatment were 1.23 ± 0.01 *F*/*F*_0_ units (*N* = 5) (**Figures 2B,D**). However, there were no significant differences in the Ca^2+^ increases (that was elicited by subsequent application of 2.5 mM extracellular Ca^2+^) between with DHPG pretreatment (1.24 ± 0.007 *F*/*F*_0_ units; *N* = 8) and without any pretreatment (**Figure 2F**).

### Synta66 and BTP2 Inhibited Store-Operated Ca^2+^ Entry (SOCE)

**FIGURE 3:**
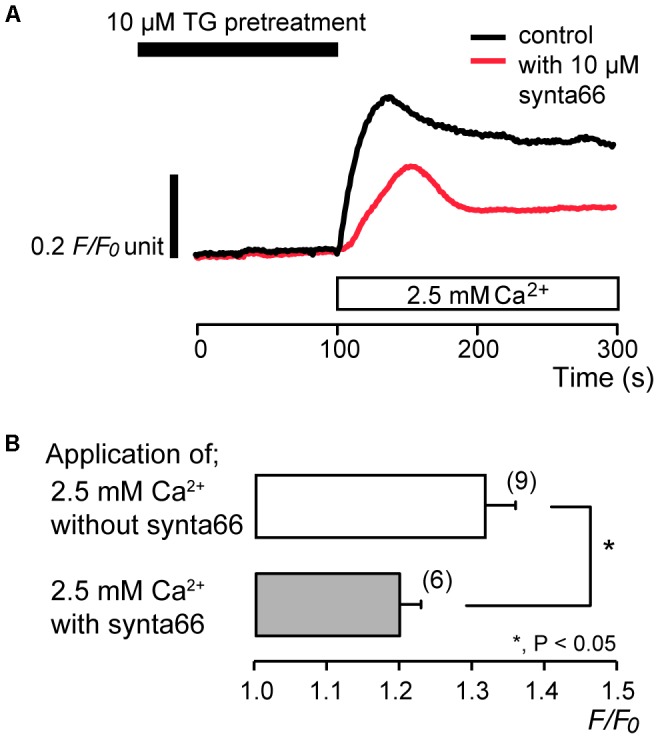
Synta66 inhibits SOCE. **(A)** Representative traces of SOCE in response to subsequent application of 2.5 mM extracellular Ca^2+^ (white box at bottom) after pre-incubation with 10 μM TG (black box at the top) with (red line), or without (black line) 10 μM synta66. **(B)** Summary bar graph shows [Ca^2+^]_i_ increases by addition of extracellular Ca^2+^ without (open column) or with 10 μM synta66 (gray column). Each column indicates the mean ± SE of 6–9 independent experiments. Statistically significant differences between columns (shown by solid lines) are denoted by asterisks, ^∗^*P* < 0.05.

**FIGURE 4:**
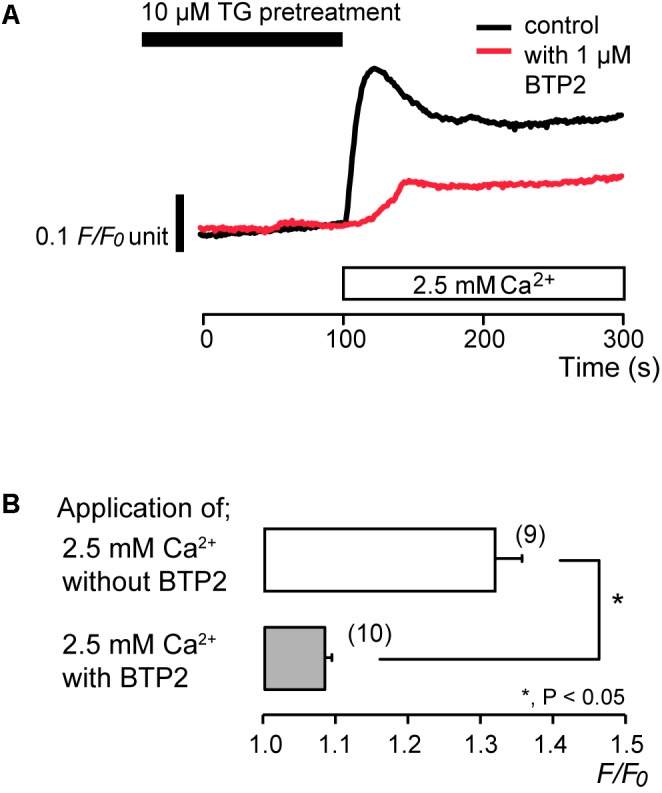
BTP2 inhibits SOCE. **(A)** Representative traces of SOCE in response to subsequent application of 2.5 mM extracellular Ca^2+^ (white box at bottom) after pre-incubation with 10 μM TG (black box at the top) with (red line), or without (black line) 1 μM BTP2. **(B)** Summary bar graph shows [Ca^2+^]_i_ increase by addition of extracellular Ca^2+^ without (open column) or with 1 μM BTP2 (gray column). Each column indicates the mean ± SE of 9–10 independent experiments. Statistically significant differences between columns (shown by solid lines) are denoted by asterisks, ^∗^*P* < 0.05.

To identify the pathway of Ca^2+^ influx activated by subsequent application of 2.5 mM extracellular Ca^2+^ after store depletion, we investigated the effects of CRAC channel inhibitors, synta66 (Beech, 2012; Kruchten et al., 2012; Derler et al., 2013; Molnár et al., 2016) and BTP2 (Ishikawa et al., 2003; Zitt et al., 2004; Zeng et al., 2017), on the Ca^2+^ influx. After store depletion by pretreatment of 10 μM TG in the absence of extracellular Ca^2+^, application of 2.5 mM extracellular Ca^2+^ increased [Ca^2+^]_i_ to a peak value of 1.32 ± 0.04 *F*/*F*_0_ units (*N* = 9). The increases in [Ca^2+^]_i_ were significantly suppressed in the presence of 10 μM synta66 to 1.20 ± 0.03 *F*/*F*_0_ units (*N* = 6) (**Figures 3A,B**). In addition, when the cells were subjected to preincubation with 1 μM BTP2 for 60 min at 37°C, the [Ca^2+^]_i_ increases following Ca^2+^ store depletion by TG pretreatment were inhibited to 1.08 ± 0.01 *F*/*F*_0_ units (*N* = 10) (**Figures 4A,B**) compared to those without BTP2 (1.32 ± 0.04 *F*/*F*_0_ units (*N* = 9).

### Lanthanum Inhibited SOCE

**FIGURE 5:**
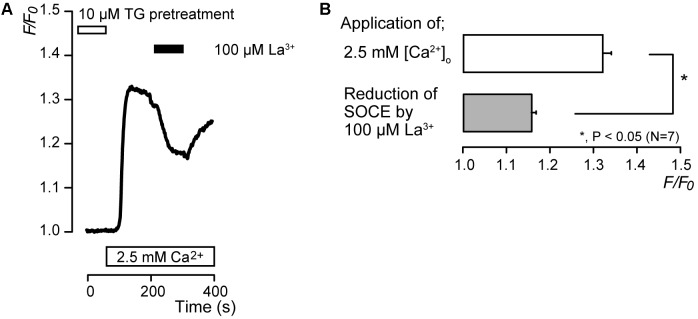
La^3+^ inhibits SOCE. **(A)** Representative trace of the effect of La^3+^ on SOCE induced by subsequent application of 2.5 mM extracellular Ca^2+^ (white box at bottom) after pre-incubation of 10 μM TG (white box at the top). Black box shows the time period of 100 μM La^3+^ addition to the extracellular solution. **(B)** Summary bar graph shows the effect of La^3+^ on SOCE (gray column) or the peak *F*/*F*_0_ values in SOCE activation (open column). Each column indicates the mean ± SE of seven independent experiments. Statistically significant differences between columns (shown by solid lines) are denoted by asterisks, ^∗^*P* < 0.05.

After store depletion by pretreatment with 10 μM TG in the absence of extracellular Ca^2+^, application of 2.5 mM extracellular Ca^2+^ increased [Ca^2+^]_i_ to a peak value of 1.32 ± 0.02 *F*/*F*_0_ units (*N* = 7), and an application of 100 μM lanthanum (La^3+^), a non-specific CRAC channel inhibitor (Ross and Cahalan, 1995; Derler et al., 2013; Guido et al., 2015; Prakriya and Lewis, 2015), caused a decrease in [Ca^2+^]_i_ to a peak value of 1.16 ± 0.01 *F*/*F*_0_ units (*N* = 7) (**Figures 5A,B**). After La^3+^-induced suppression of SOCE, removal of La^3+^ resulted in a slow return of SOCE activity over several minutes (**Figure 5A**).

### Alkaline Stimuli Enhanced SOCE

**FIGURE 6:**
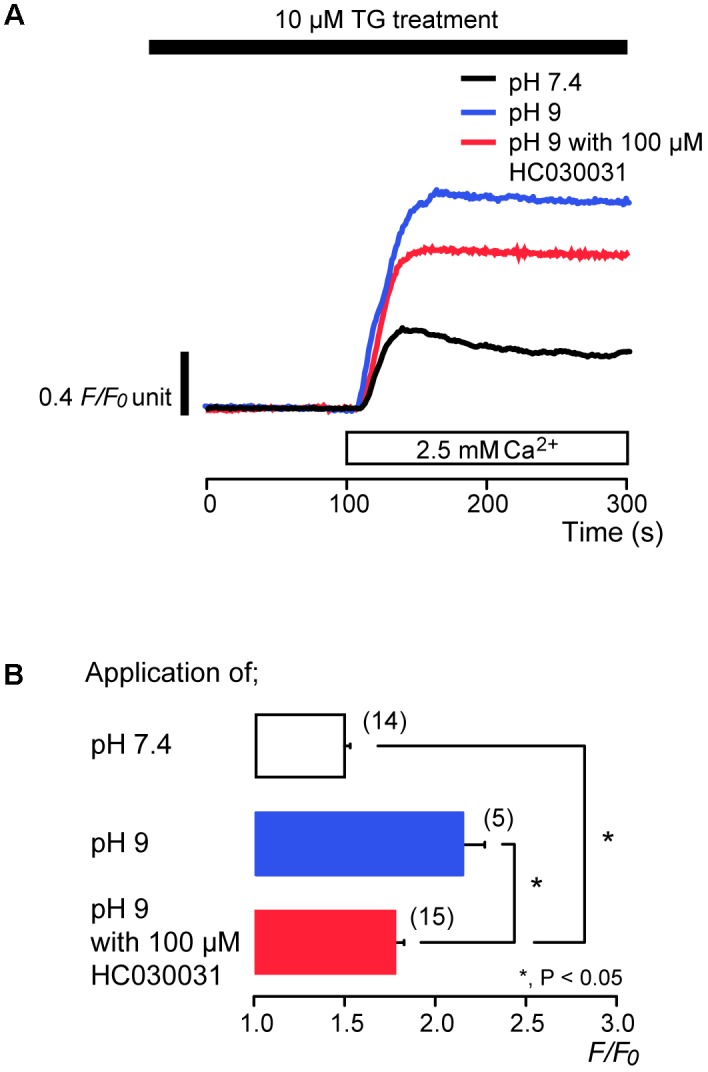
Alkaline stimuli enhance Ca^2+^ entry during TG treatment. **(A)** Representative traces of subsequent application of 2.5 mM extracellular Ca^2+^ (white box at bottom) after pre-incubation of 10 μM TG at pH 7.4 (black line) or at pH 9 without (blue line) or with (red line) 100 μM HC030031. Black box at the top indicates application of 10 μM TG. **(B)** Summary bar graph shows [Ca^2+^]_i_ increases in response to addition of extracellular Ca^2+^ at pH 7.4 (open column) or at pH 9 without (blue column) or with (red column) 100 μM HC030031. Note that the application time period of TG **(A)** was longer than that in **Figures 1**, **3**–**5**, and thus a peak value of [Ca^2+^]_i_ for the SOCE at pH 7.4 (black line in **A** and open column in **B**) was larger than that in **Figures 1**, **3**–**5**. Each column indicates the mean ± SE of 5–15 independent experiments. Statistically significant differences between columns (shown by solid lines) are denoted by asterisks, ^∗^*P* < 0.05.

We examined the effects of extracellular alkalization on SOCE in odontoblasts. After store depletion by continuous treatment with 10 μM TG in the absence of extracellular Ca^2+^, subsequent application of alkaline solution (pH 9) with 2.5 mM extracellular Ca^2+^ enhanced SOCE to a peak value of 2.17 ± 0.1 *F*/*F*_0_ units (*N* = 5) (blue; **Figures 6A,B**), while the peak value of [Ca^2+^]_i_ increase by application of standard (pH 7.4) extracellular solution with extracellular 2.5 mM Ca^2+^ was 1.49 ± 0.03 *F*/*F*_0_ units (*N* = 14) (black in **Figure 6A**). Odontoblasts express alkali-sensitive TRPA1 channels (Tsumura et al., 2013; Kimura et al., 2016). To remove the Ca^2+^ influx component via TRPA1 channel activation from SOCE by the subsequent application of alkaline solution with extracellular Ca^2+^, we applied HC030031, a TRPA1 channel antagonist (McNamara et al., 2007; Tsumura et al., 2013). HC030031 (100 μM) suppressed SOCE by the subsequent application of alkaline solution with 2.5 mM extracellular Ca^2+^ to 1.78 ± 0.04 *F*/*F*_0_ units (*N* = 15) (red; **Figures 6A,B**). The SOCE evoked by subsequent application of 2.5 mM extracellular Ca^2+^ with 100 μM HC030031 at pH 9 were larger than those without HC030031 at pH 7.4 (*N* = 14) (**Figures 6A,B**).

## Discussion

We elucidated the expression and pharmacological properties of CRAC channels in odontoblasts. After store depletion, application of extracellular Ca^2+^ induced Ca^2+^ influx (SOCE). The Ca^2+^ influx was suppressed by CRAC channel inhibitors, synta66, BTP2 and lanthanum. The activation of P2Y and muscarinic-cholinergic receptors triggered SOCE. However, the activation of group I metabotropic glutamate receptors did not elicit SOCE. After store depletion, alkaline solution containing Ca^2+^ enhanced SOCE under TRPA1 channel inhibition, compared to that using the extracellular solution with Ca^2+^ (pH 7.4) without TRPA1 inhibition. These results indicate that, in odontoblasts, store depletion activates CRAC channel-mediated SOCE, which is promoted in an alkaline environment. The results are in line with previous results showing the expression of Orai1, subunits of CRAC channels, by immunohistochemical analysis in odontoblasts (Zheng et al., 2015). CRAC channels, which mediate SOCE, are involved in various functions in diverse cells. In ameloblasts, CRAC channel-mediated SOCE contributes to the mechanism for Ca^2+^ uptake in enamel formation (Nurbaeva et al., 2015a,b). It has been also reported that dental pulp stem cells (DPSCs) express Orai1, and Orai1-knocked down shRNA suppress mineralization by DPSCs (Sohn et al., 2015). These results suggest that CRAC channel-mediated SOCE in odontoblasts has a potential role in dentinogenesis.

TRP canonical subfamily (TRPC) channels have been also proposed as possible candidates for the channel proteins mediating SOCE (Cheng et al., 2011). Among the TRPC channels, TRPC1 channels have been well-characterized and reported to participate in endogenous SOCE in several cell types (Cheng et al., 2011). Following store depletion, STIM1 translocates to the endoplasmic reticulum-plasma membrane junction and interacts with Orai1, resulting in CRAC channel activation. Ca^2+^ entry via Orai1 initiates the recruitment of TRPC1 channels into the plasma membrane, where the channels interact with STIM1 and are activated. According to this mechanism (Hogan and Rao, 2015; Ong et al., 2016; Ambudkar et al., 2017), TRPC1 channel function crucially depends on Orai1-mediated Ca^2+^ entry, and SOCE is generated by both Orai1 and STIM1, and TRPC1 channels. Odontoblasts in rats and humans have been shown to express TRPC1 channels (Kwon et al., 2014; Song et al., 2017). It has been reported that BTP2 (CRAC channel inhibitor) also inhibit SOCE via TRPC3 and TRPC5 channels (He et al., 2005). If odontoblasts express TRPC3 or/and TRPC5 channels, these channels may also contribute to SOCE in odontoblasts. Thus, TG-induced SOCE arises not only via CRAC channels but also via TRPC channel family in odontoblasts.

In the absence of extracellular Ca^2+^, P2Y, muscarinic-cholinergic, and group I metabotropic glutamate receptor agonists increased [Ca^2+^]_i_ in odontoblasts. The results demonstrate the expression of these receptors in odontoblasts, and are in line with previous reports (Shibukawa and Suzuki, 2003; Kim et al., 2009; Sato et al., 2015; Shibukawa et al., 2015; Nishiyama et al., 2016). These PLC-coupled receptors facilitate the activation of SOCE by store depletion. In odontoblasts, P2Y and muscarinic-cholinergic receptor activation elicited store depletion, resulting in the activation of SOCE. Non-excitable cells, such as microglia, cells in the adrenal cortex, and salivary gland cells, also express SOCE activated by P2Y and/or muscarinic-cholinergic receptor activations to mediate cellular functions (Nishi et al., 2013; Ambudkar, 2014; Michaelis et al., 2015). On the other hand, we could find a few reports describing SOCE activated by group I metabotropic glutamate receptor activation in hippocampal neuron and astrocytes (Ng et al., 2011; Ronco et al., 2014). The present study could not find group I metabotropic glutamate receptor-induced SOCE. Further study will be needed to clarify SOCE induced by the activation of glutamate receptors.

In the present study, alkaline stimuli enhanced SOCE via CRAC channels in odontoblasts. In our previous study, we predicted that alkaline stimuli might also activate Ca^2+^-permeable channels in addition to TRPA1 channels in odontoblasts (Kimura et al., 2016). It has been proposed that external pH modulates CRAC channel activation through its channel pore (Beck et al., 2014). In addition, external alkaline conditions augment the amplitudes of both CRAC current and [Ca^2+^]_i_ increases by SOCE (Iwasawa et al., 1997; Laskay et al., 2005; Beck et al., 2014). These evidences suggested directly regulated mechanisms for the activation of CRAC channels by alkaline stimuli. In our previous study, alkaline stimuli also elicited Ca^2+^ release from Ca^2+^ stores via metabotropic receptors in odontoblasts (Kimura et al., 2016). Although we speculated that the activation of alkali sensitive-metabotropic receptors may induce store depletion resulting in SOCE, further study will be needed to identify the molecular entity of these alkali sensitive-metabotropic receptors (Kimura et al., 2016). Odontoblasts also detect high pH produced by dental materials such as calcium hydroxide or mineral trioxide aggregate (MTA), and the alkaline stimuli increase the mineralization level in odontoblasts via TRPA1 channel activation (Kimura et al., 2016). Thus, we suggest that CRAC channel-mediated SOCE may also participate in dentinogenesis under high pH as well as physiologic conditions.

The stimuli to the dentin surface induce [Ca^2+^]_i_ increases via mechanosensitive TRP channels (Sato et al., 2015; Shibukawa et al., 2015), and Piezo channels (Sato et al., 2018) in odontoblasts. The [Ca^2+^]_i_ increases elicit the release of ATP from pannexin-1 channels (Sato et al., 2015; Shibukawa et al., 2015) in odontoblasts. The released ATP is also hydrolyzed by nucleoside triphosphate diphosphohydrolase-2 to produce ADP (Sato et al., 2015; Shibukawa et al., 2015). Therefore, ADP/ATP released from odontoblasts, as intercellular-/neuro-transmitters, also promotes Ca^2+^ signaling by the activation of ADP-induced SOCE, which involves P2Y receptor activation, in odontoblasts located in the periphery. The presence of cholinergic nerves, post-ganglionic parasympathetic fibers, in the dental pulp is controversial. If post-ganglionic parasympathetic nerves innervate the dental pulp, acetylcholine released by excitation of the neurons could activate SOCE in odontoblasts.

**FIGURE 7:**
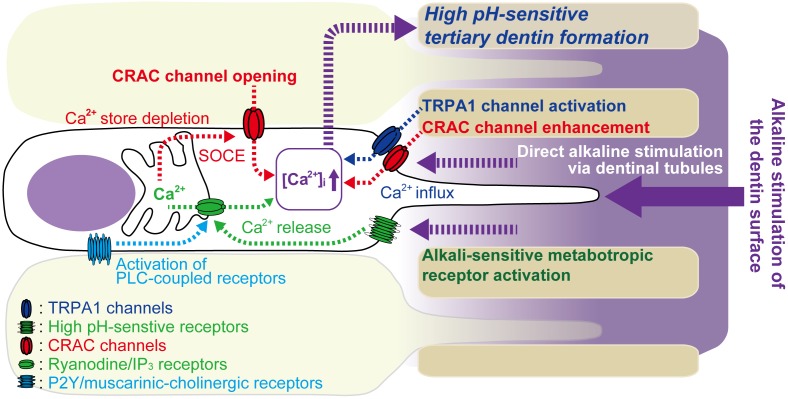
Schematic of Ca^2+^ signaling pathway activated by alkaline stimuli to dentin surface and PLC-coupled receptor activation in odontoblasts. Odontoblasts express PLC-coupled P2Y and muscarinic-cholinergic receptors. P2Y receptors are activated by endogenous ATP/ADP released from odontoblasts in the dental pulp in response to cellular deformation or cellular damage following dentin stimuli. Muscarinic-cholinergic receptors are activated by agonist released from intradental parasympathetic neurons. Activation of these receptors induces depletion of Ca^2+^ stores by intracellular Ca^2+^ release from intracellular Ca^2+^ stores via ryanodine and/or IP_3_ receptors, resulting in increased [Ca^2+^]_i_. Ca^2+^ store depletion then activates store-operated Ca^2+^ entry (SOCE) via CRAC channels in odontoblasts. Direct alkaline stimuli via dentinal tubules to odontoblasts modulate CRAC channel activation and enhance Ca^2+^ influx via CRAC channels. The alkaline stimuli also activate TRPA1 channels in odontoblasts and evoke Ca^2+^ influx via TRPA1 channels. Additionally, alkaline stimuli activate alkali sensitive-metabotropic receptors, inducing Ca^2+^ release from intracellular Ca^2+^ stores via ryanodine and/or IP_3_ receptors. This Ca^2+^ release may, in turn, induce store depletion and SOCE via CRAC channels in odontoblasts. These high-pH-sensitive Ca^2+^ signaling pathways may play important roles in tertiary dentin formation by odontoblasts, following application of alkali stimuli, such as high-pH dental materials, on the dentin surface.

In conclusion (see **Figure 7**), we demonstrated SOCE mediated by CRAC channels in odontoblasts. SOCE is activated by PLC-coupled receptors in odontoblasts. Endogenous ADP, released from odontoblasts in the dental pulp in response to cellular deformation or cellular damage, as well as muscarinic-cholinergic agonist from intradental parasympathetic neurons, evoked SOCE in odontoblasts. SOCE was enhanced by an alkaline environment and may play important roles in accelerating cellular functions, such as high-pH sensitive tertiary/reactionary dentin formation, following alkaline stimuli applied to dentin. In addition, alkaline stimuli activate TRPA1 channels in odontoblasts and evoke Ca^2+^ influx via TRPA1 channels. [Ca^2+^]_i_ increases due to TRPA1 channel-mediated Ca^2+^ influx, which is closely involved in dentin formation under both physiological and high pH conditions (Kimura et al., 2016). Alkaline stimuli also activate alkali sensitive-metabotropic receptors (Kimura et al., 2016), and their activation induces Ca^2+^ release from intracellular Ca^2+^ stores via ryanodine and/or IP_3_ receptors. During dental treatments, the use of dental materials, such as calcium hydroxide or MTA, results in a high pH/Ca^2+^ extracellular environment. This external environment activates Ca^2+^ signaling mediated by SOCE, TRPA1 channels and alkali sensitive-metabotropic receptors, which can sense the increased pH in odontoblasts, resulting in the induction of dentinogenesis.

## Author Contributions

MK, KN, MT, and YS were responsible for the conception and design of the experiments. MK, KN, AH, SO, KS, MT, and YS were responsible for the acquisition, analysis, and interpretation of data. MK, KN, and YS were responsible for drafting and critically revising the intellectual content of the article. YS was responsible for final approval of the version to be submitted/published. All authors read and approved the final manuscript.

## Conflict of Interest Statement

The authors declare that the research was conducted in the absence of any commercial or financial relationships that could be construed as a potential conflict of interest.
